# Supplementary material for: Fibrinolytic-deficiencies predispose hosts to septicemia from a catheter-associated UTI
Source: Nat Commun. 2024 Mar 27;15:2704. doi: 10.1038/s41467-024-46974-6 (PMC10973455; doi:10.1038/s41467-024-46974-6)
Supplement: Supplementary file 1 — Supplementary Information [file 41467_2024_46974_MOESM1_ESM.pdf]

Supplementary Information

Fibrinolytic-deficiencies predispose hosts to septicemia from a catheter-associated UTI.

Jonathan J. Molina[1,2,10], Kurt N. Kohler[2,10], Christopher Gager[2], Marissa J. Andersen[2], Ellsa Wongso[2], Elizabeth R. Lucas[2], Andrew Paik[2], Wei Xu[5,6], Deborah Donahue[3,4], Karla Bergeron[7], Aleksandra Klim[7], Michael G. Caparon[5,6], Scott J. Hultgren[5,6], Alana Desai[7], Victoria A. Ploplis [3,4], Matthew J. Flick[8,9], Francis J. Castellino[3,4], and Ana L. Flores-Mireles[1,2,3,11,*].

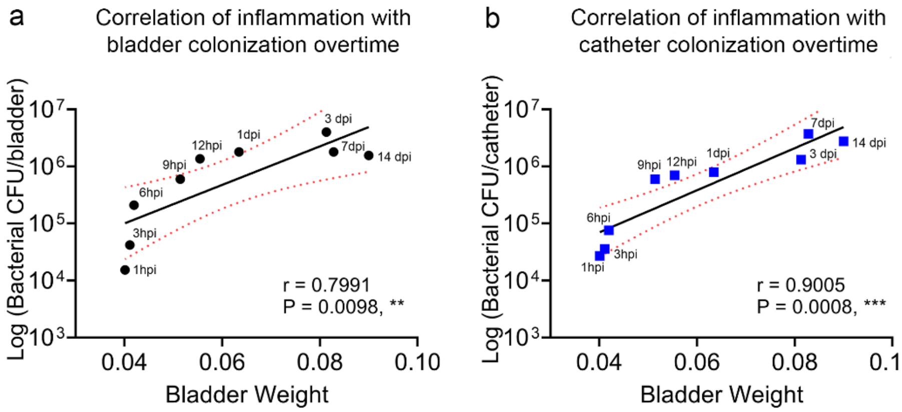

Supplementary Figure 1. Catheter-induced inflammation correlates with pathogen colonization overtime. Pearson's correlation statistical analysis was used to quantify the association between the catheter-induced bladder inflammation and pathogen colonization in the bladder (a) or catheter (b) overtime. For this test, median bladder weights values from catheterized and infected mice and the median values of the CFUs of bladders or catheters were used to perform this test. r, Pearson’s correlation coefficient. *, $P < 0.05$ was considered statistically significant. **, $P < 0.005$; ***, $P < 0.0005$. Data provided in Source Data file.

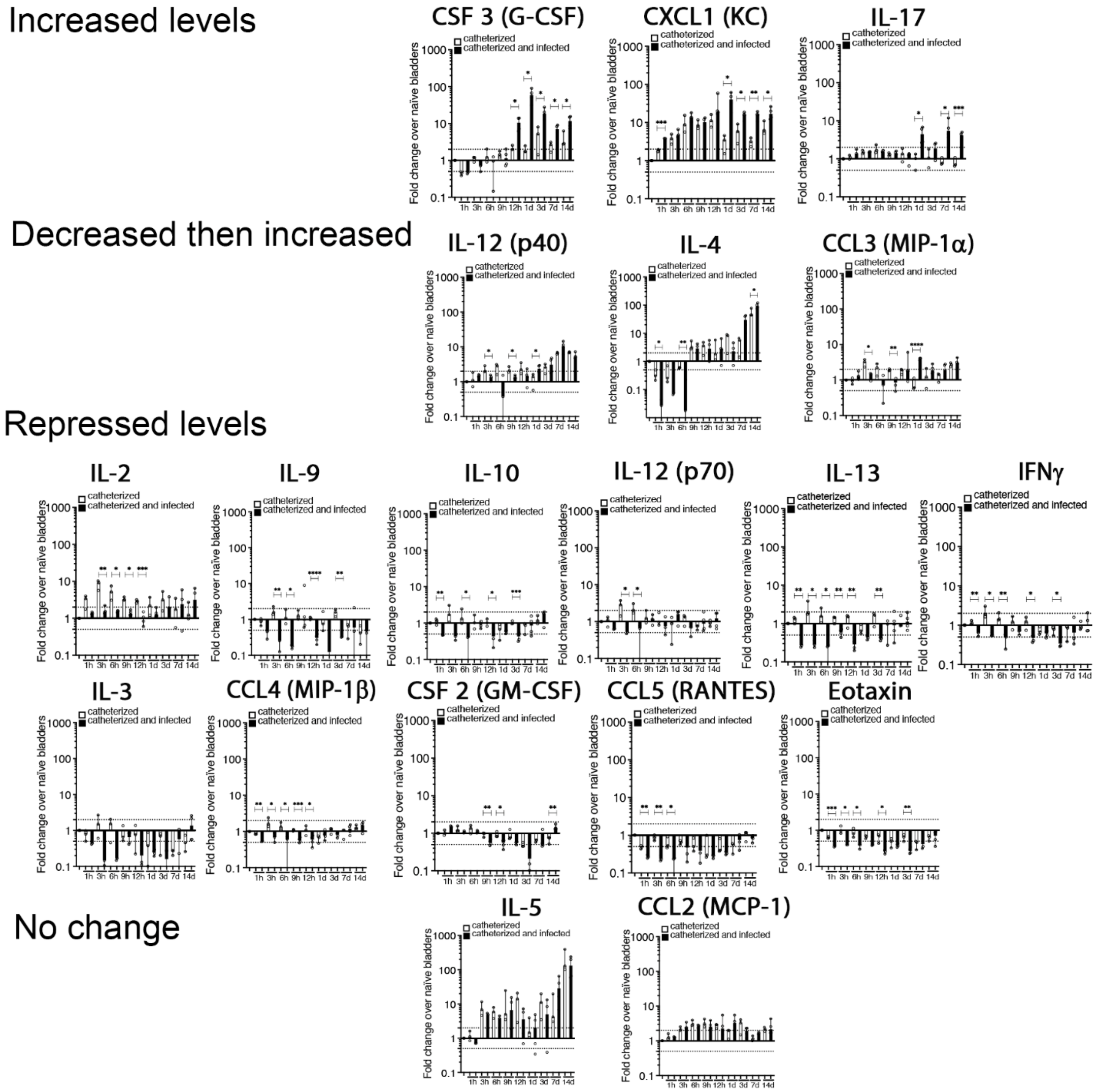

Supplementary Figure 2. Inflammatory cytokines of the catheterized bladder. Analysis of individual cytokine levels in the catheterized bladder (with or without *E. faecalis* infection) that increased, decreased then decreased, were repressed, or had no change. The horizontal broken line represents 2-fold change cytokine levels compared with naïve control mice. The horizontal bar represents the median value with range as error bars. The Mann-Whitney U test was used; *, P < 0.05 was considered statistically significant. **, P < 0.005; ***, P < 0.0005; ****, P<0.0001. For all graphs, n=3-4 depending on time points and exact n and source data is provided in Source Data file. Animals that lost the catheter were not included in this work.

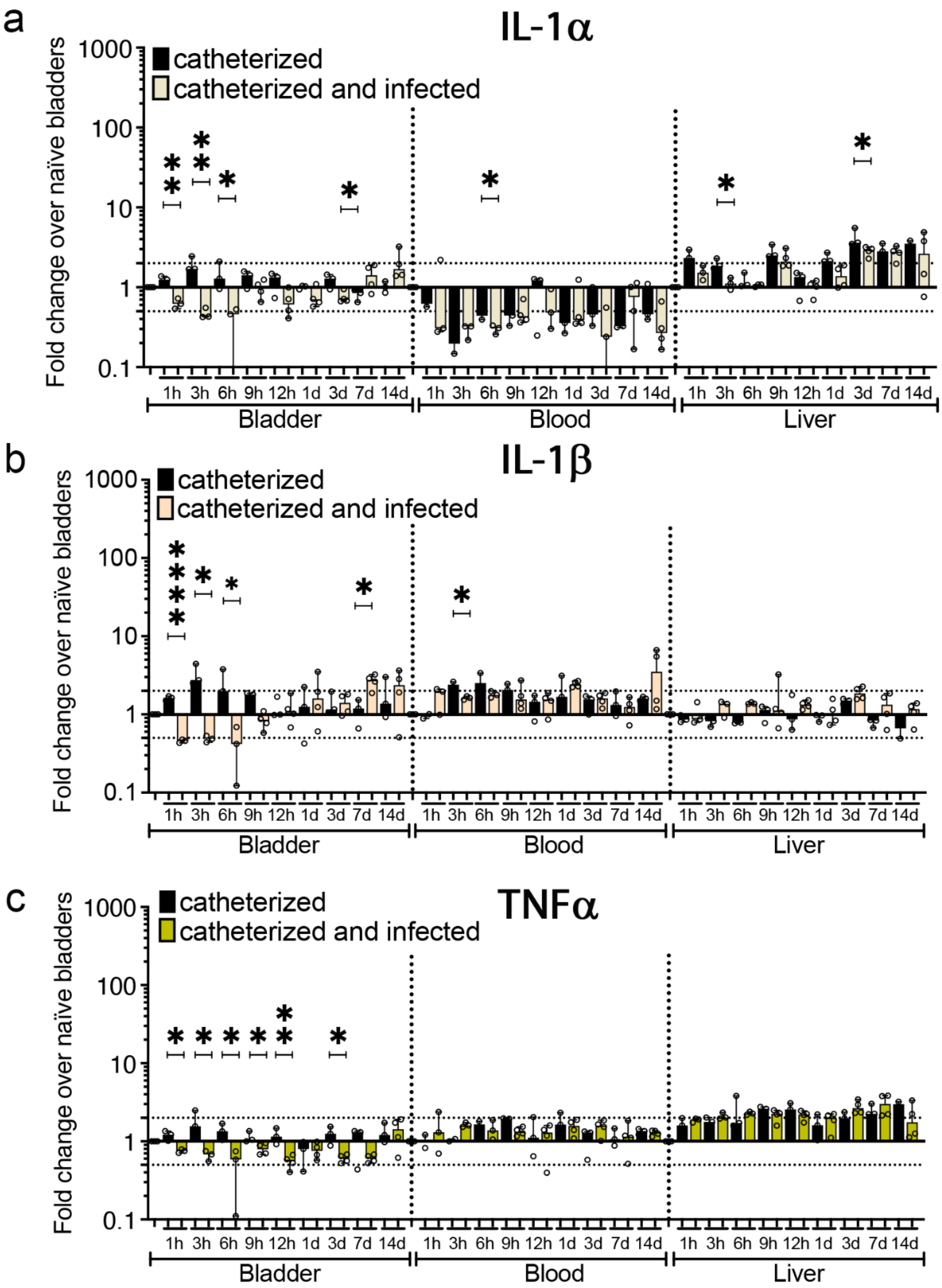

Supplementary Figure 3. Comparative analysis of 1L-1α, IL-1β, and TNFα levels. 1L-1α (a), IL-1β (b), and TNFα (c) levels in the catheterized bladder (with or without infection), bloodstream, and liver. The

horizontal broken line represents twofold change cytokine levels compared with naïve control mice over a 2-fold change. The horizontal bar represents the median value with range as error bars. The Mann-Whitney U test was used; *, $P < 0.05$ was considered statistically significant. **, $P < 0.005$; ****, $P<0.0001$. For all graphs, n=3-4 depending on time points. Source data and exact n is provided in Source Data file. Animals that lost the catheter were not included in this work.

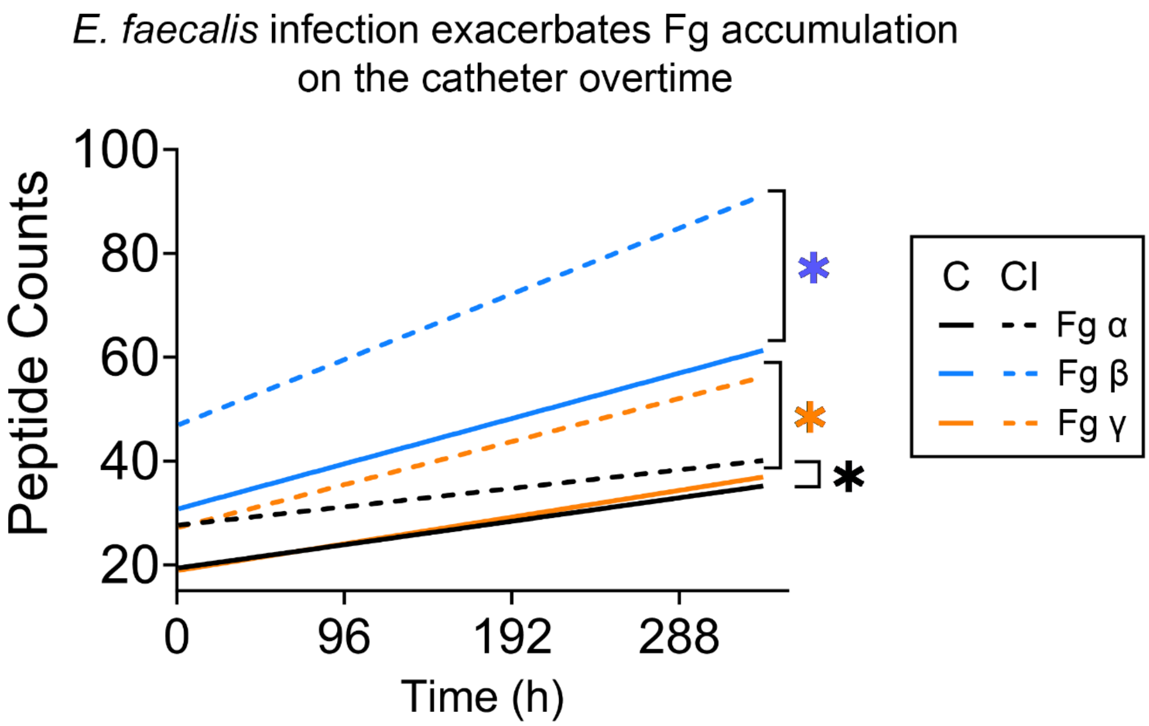

Supplementary Fig. 4. *E. faecalis* infection significantly increased Fg accumulation on urinary catheters overtime. Line graph of total Fg-α, -ß, and -γ peptide counts found on catheters of mice catheterized and infected with *E. faecalis* (CI) or catheterized and mock-infected (C) over a temporal study from naïve (0 hrs) to 14 days. Simple linear regressions were conducted to determine the association lines between time and total peptide counts of all three peptide counts from C and CI cohorts. Then, an F-test was conducted to quantify the difference between lines from C and CI when analyzing Fg-α, Fg-β, and Fg-γ peptide counts: Fg-α ($F=5.039$, $p=0.0403$); Fg-β ($F=5.110$, $p=0.0391$); Fg-γ ($F=6.386$, $p=0.0232$). *, n=9 for each line on graph and $P < 0.05$ was considered statistically significant. Source data is provided in Source Data file.

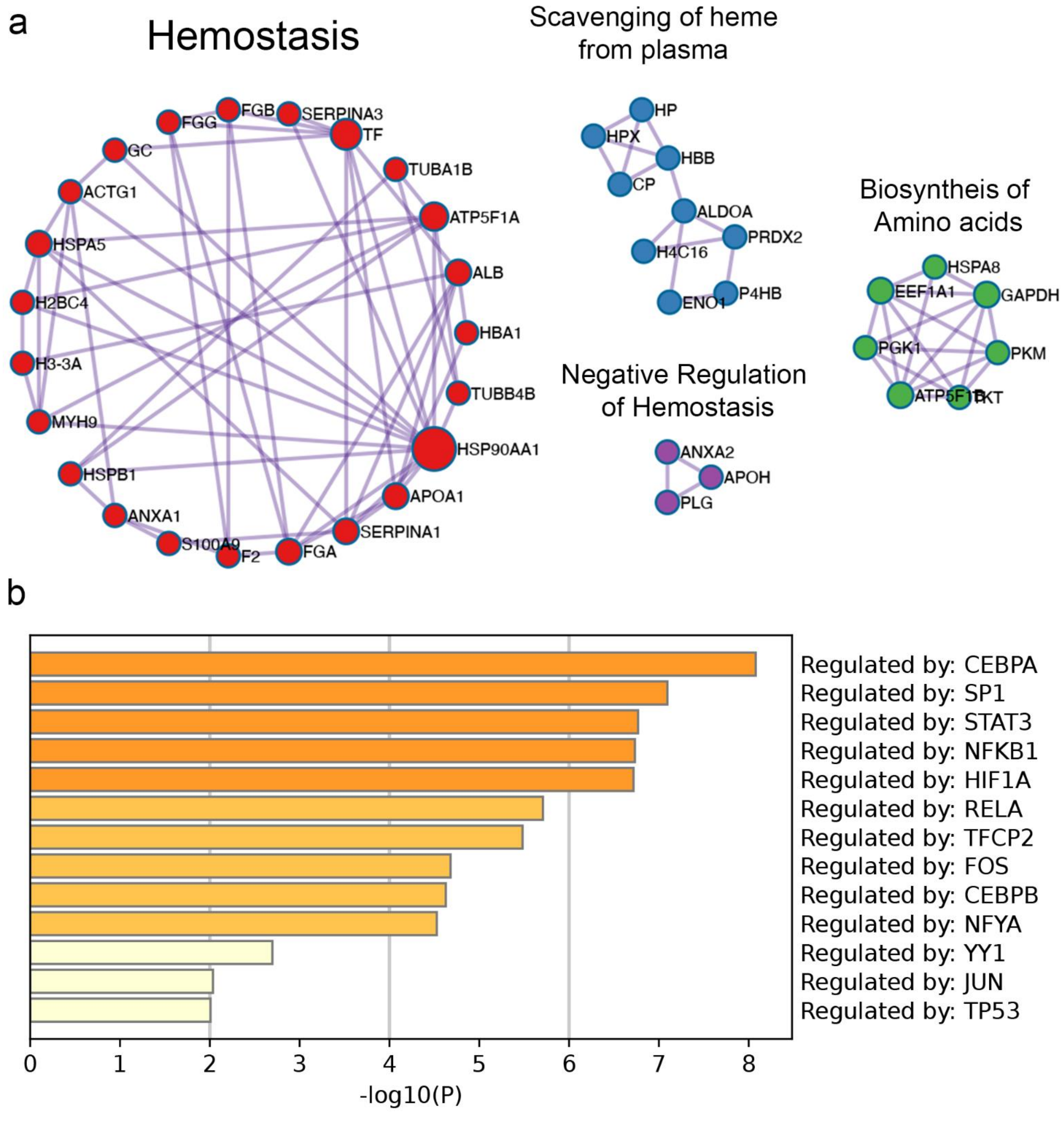

Supplementary Figure 5. Protein-protein interactions and summary of transcriptional regulatory analysis. 76 shared mouse and human catheter proteins from Fig. 2 were analyzed on metascape.org[1]. (a) Molecular Complex Detection (MCODE) components identified from protein-protein interaction

enrichment analysis. Generated networks were modified using Cytoscape network analysis and clusters of interactions were labelled by the top descriptive gene ontology for each protein-protein interaction[2]. (b) Summary of enrichment analysis of transcriptional regulatory relationships unraveled by sentence base text mining (TRRUST)[3]. Colors are used to distinguish individual networks from one another. Top Predicted transcriptional regulators were plotted based on -log10(P) values as provided by Metascape without modifications.

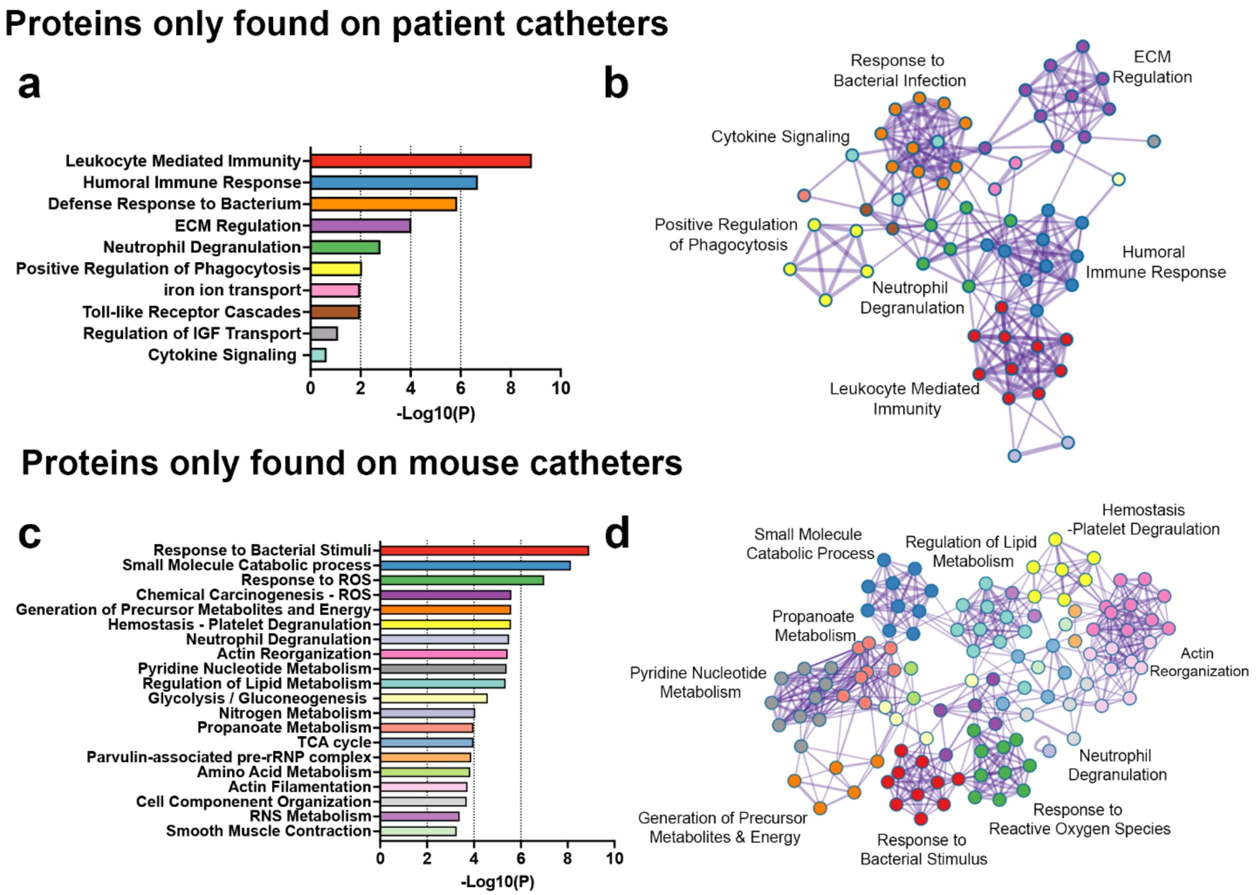

Supplementary Fig. 6. Protein pathways of the differential deposited proteins on urinary catheters retrieved from patients and mice. Independent network and pathway analyses using Metascape were done on proteins only found on either mouse or patient catheters. Metascape analysis showing the top significant pathways in proteins found only on human catheters (a-b) or on mouse catheters (c-d). Metascape network analysis and clusters of interactions of the top significant pathways were visualized with Cytoscape (b,d). Source data is provided in Source Data file.

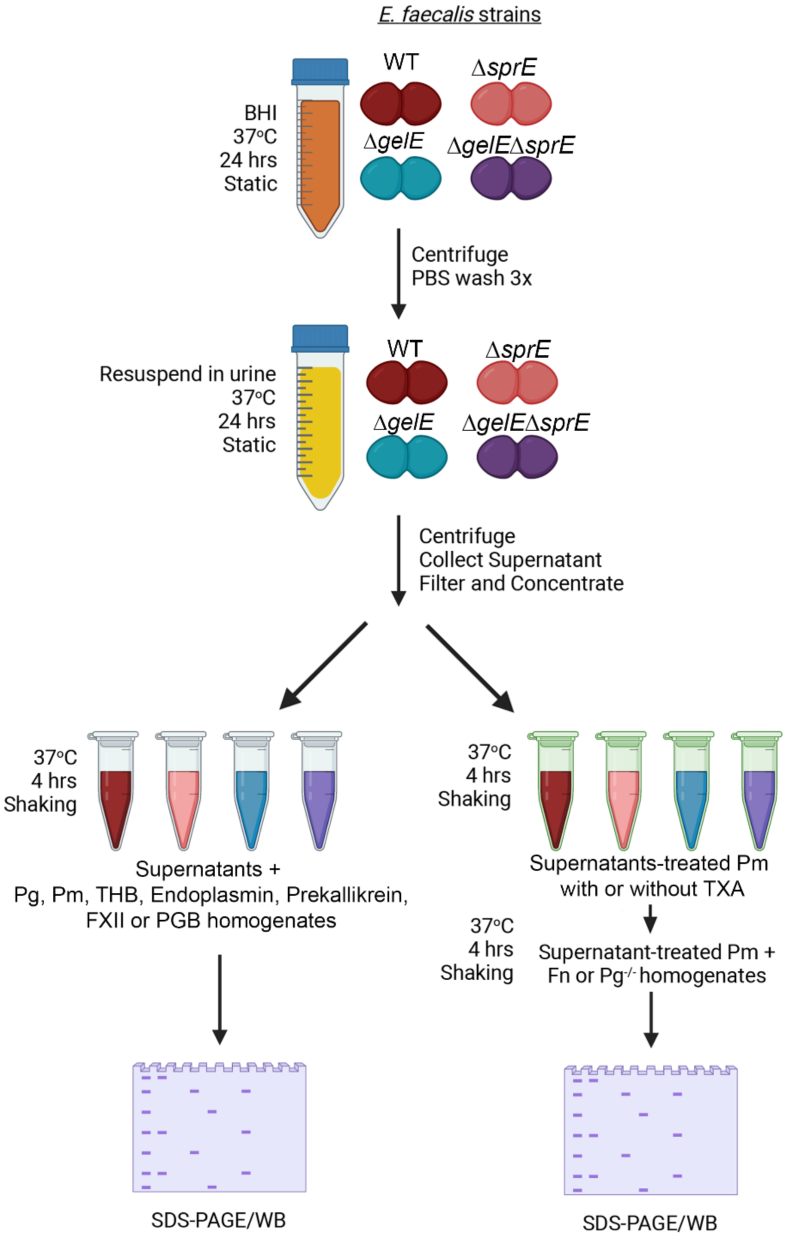

Supplementary Fig. 7. Experimental procedure. Graphical illustration of methodology for Fig. 4 and Supplementary Fig. 8. *E faecalis* WT and protease mutant strains were grown in BHI then transferred to urine. Supernatants *E. faecalis* grown in urine were filtered and concentrated, then incubated with plasminogen (Pg), plasmin (Pm), thrombin (THB), Endoplasmin, Prekallikrein, or Factor XII (FXII) purified proteins or PGB or $Pg^{-/-}$ mouse bladder homogenates from 24 hrs catheterized non-infected mice.

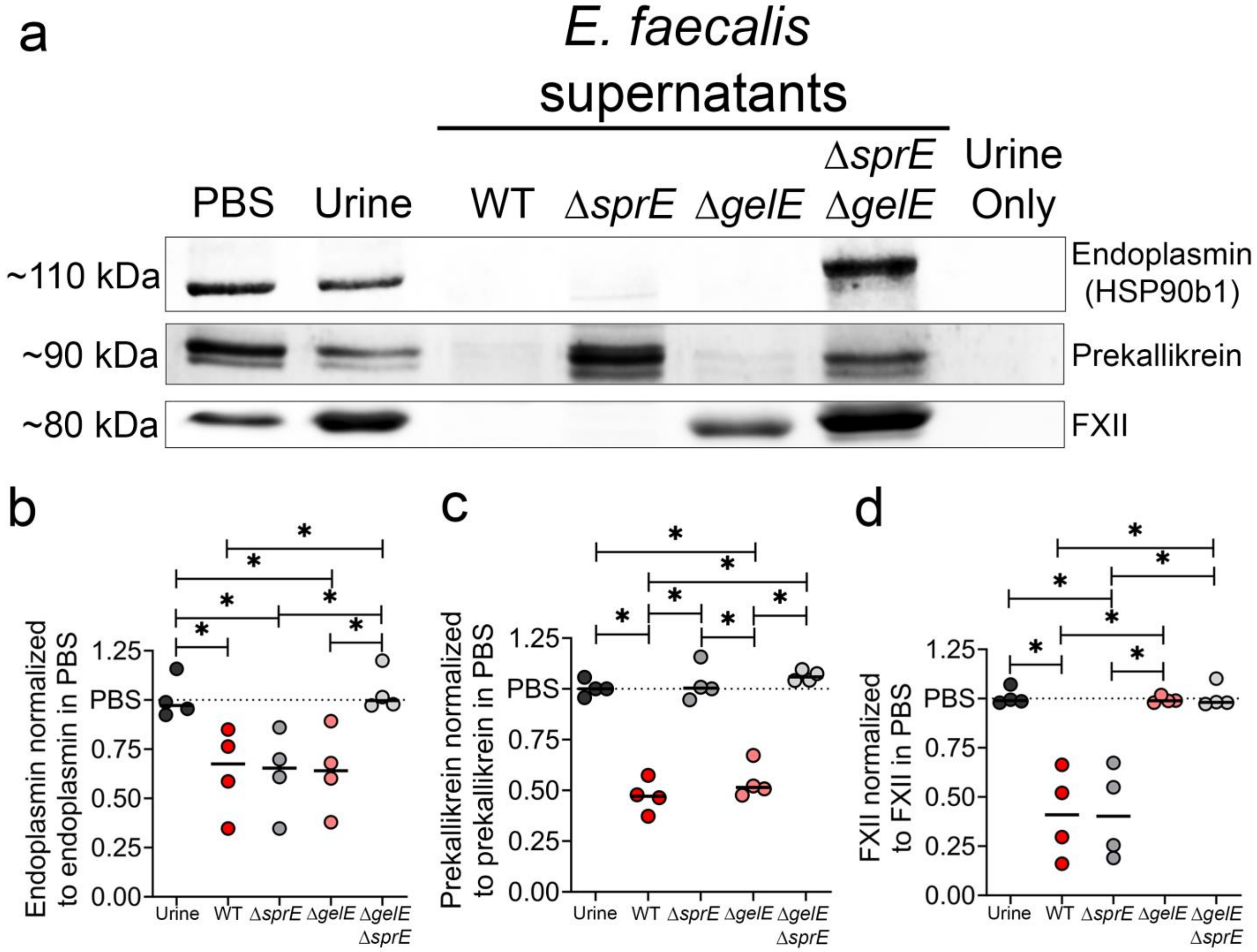

Supplementary Figure 8. SDS-PAGE analysis of the proteolytic activity of *E. faecalis* WT and protease mutants' cell-free supernatants against different purified human proteins. (a) Endoplasmin, Prekallikrein, or Factor XII (FXII) and their corresponding degradation quantification by densitometry after 4 repeats of each blot (b-d). Values represent the median. The Mann-Whitney U test was used to determine significance; *, $P < 0.05$ was considered statistically significant. The horizontal bar represents the median value and b-d) n=4. Source data and uncropped images included in source data file.

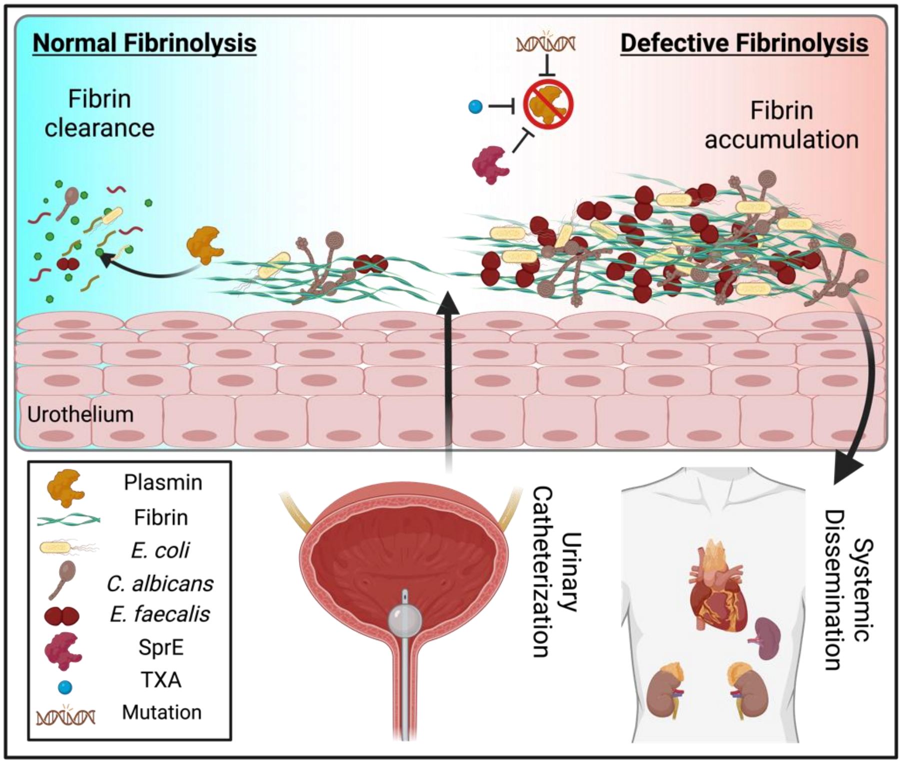

Supplementary Figure 9. Fibrinolytic-deficiencies predispose hosts to severe CAUTI and septicemia by diverse uropathogens in mono- and poly-microbial infections. Defective fibrinolysis drives extravascular fibrin formation predisposing hosts to severe catheter associated urinary tract infections and systemic dissemination by prevalent CAUTI pathogens. *E. coli*, *E. faecalis*, and *C. albicans* each utilize fibrin for biofilm formation and virulence. Defective fibrinolysis results from congenital deficiencies, pharmacological inhibition through tranexamic acid, or by pathogen-secreted proteases such as *E. faecalis*' serine protease SprE.

Supplementary Table 1. Top 20 clusters of proteins found on both human and mouse catheters with their representative enriched terms (one per cluster) analyzed with Metascape[1] using a hypergeometric test and Benjamini-Hochberg p-value correction.

GO	Category	Description	Count	%	Log10(P)	Log10(q)
R-HSA-114608	Reactome Gene Sets	Platelet degranulation	20	26.32	-30.12	-25.77
R-HSA-6798695	Reactome Gene Sets	Neutrophil degranulation	23	30.26	-22.70	-19.05
hsa04610	KEGG Pathway	Complement and coagulation cascades	14	18.42	-21.35	-17.78
GO:0050878	GO Biological Processes	regulation of body fluid levels	19	25.00	-19.71	-16.26
WP3888	WikiPathways	VEGFA-VEGFR2 signaling	19	25.00	-17.97	-14.66
WP15	WikiPathways	Selenium micronutrient network	12	15.79	-17.47	-14.20
GO:0006959	GO Biological Processes	humoral immune response	15	19.74	-16.91	-13.72
GO:0030193	GO Biological Processes	regulation of blood coagulation	11	14.47	-16.72	-13.62
GO:0030162	GO Biological Processes	regulation of proteolysis	20	26.32	-15.10	-12.14
WP4629	WikiPathways	Aerobic glycolysis	6	7.89	-12.73	-9.92
R-HSA-2262752	Reactome Gene Sets	Cellular responses to stress	17	22.37	-11.05	-8.45
R-HSA-977606	Reactome Gene Sets	Regulation of Complement cascade	7	9.21	-10.56	-8.01
hsa04613	KEGG Pathway	Neutrophil extracellular trap formation	10	13.16	-10.30	-7.76
GO:0098869	GO Biological Processes	cellular oxidant detoxification	8	10.53	-10.27	-7.74
GO:0051702	GO Biological Processes	biological process involved in interaction with symbiont	8	10.53	-9.20	-6.72
R-HSA-9613829	Reactome Gene Sets	Chaperone Mediated Autophagy	5	6.58	-8.65	-6.24
CORUM:6823	CORUM	CPu2013LFu2013MPO complex	3	3.95	-7.82	-5.45
GO:0071345	GO Biological Processes	cellular response to cytokine stimulus	13	17.11	-7.49	-5.19
hsa05130	KEGG Pathway	Pathogenic Escherichia coli infection	8	10.53	-7.44	-5.15
GO:0035966	GO Biological Processes	response to topologically incorrect protein	7	9.21	-7.14	-4.88

GO: gene ontology

Count: is the number of genes in the user-provided lists with membership in the given ontology term.

%: is the percentage of all of the user-provided genes that are found in the given ontology term (only input genes with at least one ontology term annotation are included in the calculation).

Log10(P): is the p-value in log base 10.

Log10(q): is the multi-test adjusted p-value in log base 10.

Supplementary Table 2. Summary of enrichment analysis in TRRUST from proteins found on both humans and mouse catheters (transcriptional regulatory relationships unraveled by sentence-based text-mining)[3] determined by Metascape using a hypergeometric test and Benjamini-Hochberg p-value correction.

GO	Description	Count	%	Log10(P)	Log10(q)
TRR00109	Regulated by: CEBPA	6	7.90	-8.10	-6.00
TRR01256	Regulated by: SP1	11	14.00	-7.10	-5.10
TRR01277	Regulated by: STAT3	7	9.20	-6.80	-4.80
TRR00875	Regulated by: NFKB1	9	12.00	-6.70	-4.80
TRR00484	Regulated by: HIF1A	6	7.90	-6.70	-4.80
TRR01158	Regulated by: RELA	8	11.00	-5.70	-3.90
TRR01404	Regulated by: TFCP2	3	3.90	-5.50	-3.70
TRR00342	Regulated by: FOS	4	5.30	-4.70	-3.00
TRR00110	Regulated by: CEBPB	4	5.30	-4.60	-2.90
TRR00881	Regulated by: NFYA	3	3.90	-4.50	-2.90
TRR01548	Regulated by: YY1	3	3.90	-2.70	-1.30
TRR00645	Regulated by: JUN	3	3.90	-2.00	-0.69
TRR01419	Regulated by: TP53	3	3.90	-2.00	-0.67

GO: gene ontology

Count: is the number of genes in the user-provided lists with membership in the given ontology term.

%: is the percentage of all of the user-provided genes that are found in the given ontology term (only input genes with at least one ontology term annotation are included in the calculation).

Log10(P): is the p-value in log base 10.

Log10(q): is the multi-test adjusted p-value in log base 10.

Supplementary Table 3. Situations that may promote clotting and susceptibility to CAUTIs

	Situations that may promote clotting and susceptibility to CAUTIs					
	Hemostatic Phenotype	Inherited Diseases	Acquired disorders	Treatments	In this study	Susceptible
Fibrinolytic Pathway	Hypofibrinolysis	Congenital Pg deficiency	Pg deficiencies during Liver disease	Tranexamic Acid (TXA)	Pg$^{-/-}$	Yes
			-Liver Disease			
			-Disseminated Intravascular Coagulation (DIC)			
		Congenital uPA deficiency	PAI-1 overexpression during: metabolic syndrome cancer surgery pregnancy Inflammation (sepsis)	Aminocaproic acid (EACA)	PGB	Yes
		Congenital tPA deficiency			TXA	Yes
					tPA$^{-/-}$	No
					uPA$^{-/-}$	Yes
	Hyperfibrinolysis	PAI-1 deficiency	Liver Disease	alpha-2-plasmin inhibitor	PAI-1$^{-/-}$	No
		TAFI deficiency	Trauma			
			Disseminated Intravascular Coagulation			
			Cancer			
			Heat Stroke			
Intrinsic/Extrinsic/Common Pathway	Defective Clotting	Hemophilia A/B	Acquired hemophilia A/B,	Thrombolytic therapy	Fg$^{-/-}$	No
		von Willebrand Disease			Fg^{AEK}	No
		Factor I (Fg) deficiency			FVII^{tTA}	No
		Factor II (prothrombin) deficiency			FXII$^{-/-}$	No
					PK$^{-/-}$	No
					KNG$^{-/-}$	No
	Excessive Clotting	Thrombophilia due to: Factor V Leiden	Disseminated Intravascular Coagulation	Factor IX products	N/A	Likely
		Antithrombin deficiency	Antiphospholipid antibody syndrome	Vitamin K (promotes coagulation factors)		
		Protein C deficiency	Vitamin B6, B12, Folate deficiency	Epinephrine		
		Protein Z deficiency		Factor VIIa products		
		Prothrombin 20210 mutation (Factor II Mutation)		Factor VIII products		

Supplementary Table 4. Strains used in this study.

Species	Strain	Description	Reference
Enterococcus faecalis	OG1RF WT	Expresses both GelE and SprE	[4,5]
Enterococcus faecalis	OG1RF Δ*gelE*	Expresses SprE	[5,6]
Enterococcus faecalis	OG1RF Δ*sprE*	Expresses GelE	[5]
Enterococcus faecalis	OG1RF Δ*gelE* Δ*sprE*	No protease activity	[5]
Escherichia coli	UTI89 WT	HK::GFP	[7-9]
Candida albicans	SC5314 WT		[10]

Supplementary References

1 Zhou, Y. *et al.* Metascape provides a biologist-oriented resource for the analysis of systems-level datasets. *Nat Commun* **10**, 1523 (2019). https://doi.org:10.1038/s41467-019-09234-6

2 Shannon, P. *et al.* Cytoscape: a software environment for integrated models of biomolecular interaction networks. *Genome Res* **13**, 2498-2504 (2003). https://doi.org:10.1101/gr.1239303

3 Han, H. *et al.* TRRUST v2: an expanded reference database of human and mouse transcriptional regulatory interactions. *Nucleic Acids Res* **46**, D380-D386 (2018). https://doi.org:10.1093/nar/gkx1013

4 Murray, B. E. *et al.* Generation of restriction map of Enterococcus faecalis OG1 and investigation of growth requirements and regions encoding biosynthetic function. *J Bacteriol* **175**, 5216-5223 (1993). https://doi.org:10.1128/jb.175.16.5216-5223.1993

5 Xu, W. *et al.* Host and bacterial proteases influence biofilm formation and virulence in a murine model of enterococcal catheter-associated urinary tract infection. *NPJ Biofilms Microbiomes* **3**, 28 (2017). https://doi.org:10.1038/s41522-017-0036-z

6 Thomas, V. C. *et al.* A fratricidal mechanism is responsible for eDNA release and contributes to biofilm development of Enterococcus faecalis. *Molecular Microbiology* **72**, 1022-1036 (2009). https://doi.org:10.1111/j.1365-2958.2009.06703.x

7 Andersen, M. J. *et al.* Inhibiting host-protein deposition on urinary catheters reduces associated urinary tract infections. *Elife* **11** (2022). https://doi.org:10.7554/eLife.75798

8 Guiton, P. S. *et al.* Combinatorial small-molecule therapy prevents uropathogenic Escherichia coli catheter-associated urinary tract infections in mice. *Antimicrob Agents Chemother* **56**, 4738-4745 (2012). https://doi.org:10.1128/AAC.00447-12

9 Bi, X. C. *et al.* Pathogen incidence and antibiotic resistance patterns of catheter-associated urinary tract infection in children. *J Chemother* **21**, 661-665 (2009). https://doi.org:10.1179/joc.2009.21.6.661

10 Nobile, C. J. *et al.* A recently evolved transcriptional network controls biofilm development in Candida albicans. *Cell* **148**, 126-138 (2012). https://doi.org:10.1016/j.cell.2011.10.048
