# Supplementary material for: Fibrinolytic-deficiencies predispose hosts to septicemia from a catheter-associated UTI
Source: Nat Commun. 2024 Mar 27;15:2704. doi: 10.1038/s41467-024-46974-6 (PMC10973455; doi:10.1038/s41467-024-46974-6)
Supplement: Supplementary file 3 — Description of Additional Supplementary Files [file 41467_2024_46974_MOESM3_ESM.pdf]

Description of Additional Supplementary Files

Supplementary Data 1: Proteomic analysis of mouse catheters. This data is in an excel file.

Supplementary Data 2: Proteomic analysis of human catheters. This data is in an excel file.
