# Supplementary material for: Fibrinolytic-deficiencies predispose hosts to septicemia from a catheter-associated UTI
Source: Nat Commun. 2024 Mar 27;15:2704. doi: 10.1038/s41467-024-46974-6 (PMC10973455; doi:10.1038/s41467-024-46974-6)
Supplement: Supplementary file 6 — Reporting Summary [file 41467_2024_46974_MOESM6_ESM.pdf]

nature portfolio

Corresponding author(s): Ana L. Flores-Mireles

Last updated by author(s): Feb 27, 2024

Reporting Summary

Nature Portfolio wishes to improve the reproducibility of the work that we publish. This form provides structure for consistency and transparency in reporting. For further information on Nature Portfolio policies, see our Editorial Policies and the Editorial Policy Checklist.

Statistics

For all statistical analyses, confirm that the following items are present in the figure legend, table legend, main text, or Methods section.

n/a	Confirmed	
☐	☒	The exact sample size (n) for each experimental group/condition, given as a discrete number and unit of measurement
☐	☒	A statement on whether measurements were taken from distinct samples or whether the same sample was measured repeatedly
☐	☒	The statistical test(s) used AND whether they are one- or two-sided<br>*Only common tests should be described solely by name; describe more complex techniques in the Methods section.*
☐	☒	A description of all covariates tested
☐	☒	A description of any assumptions or corrections, such as tests of normality and adjustment for multiple comparisons
☐	☒	A full description of the statistical parameters including central tendency (e.g. means) or other basic estimates (e.g. regression coefficient) AND variation (e.g. standard deviation) or associated estimates of uncertainty (e.g. confidence intervals)
☐	☒	For null hypothesis testing, the test statistic (e.g. F, t, r) with confidence intervals, effect sizes, degrees of freedom and P value noted<br>*Give P values as exact values whenever suitable.*
☒	☐	For Bayesian analysis, information on the choice of priors and Markov chain Monte Carlo settings
☒	☐	For hierarchical and complex designs, identification of the appropriate level for tests and full reporting of outcomes
☐	☒	Estimates of effect sizes (e.g. Cohen's d, Pearson's r), indicating how they were calculated

Our web collection on statistics for biologists contains articles on many of the points above.

Software and code

Policy information about availability of computer code

Data collection	ImageStudio (Li-Cor)<br>Bio-plex Data Pro software (Bio-Rad)<br>Cytoscape (www.Cytoscape.org)<br>Evenn Network (www.ehbio.com)<br>Metascape (www.metascape.org)<br>Zen Pro (Zeiss)<br>Mascot (Matrix Science)
Data analysis	Graphpad Prism 9 was used to perform all data analysis.

For manuscripts utilizing custom algorithms or software that are central to the research but not yet described in published literature, software must be made available to editors and reviewers. We strongly encourage code deposition in a community repository (e.g. GitHub). See the Nature Portfolio guidelines for submitting code & software for further information.

Data

Policy information about availability of data

All manuscripts must include a data availability statement. This statement should provide the following information, where applicable:

- Accession codes, unique identifiers, or web links for publicly available datasets
- A description of any restrictions on data availability
- For clinical datasets or third party data, please ensure that the statement adheres to our policy

Proteomics raw data are available in Supplementary Data File 1 and 2. The mass spectrometry proteomics data have been deposited to the ProteomeXchange Consortium via the PRIDE partner repository with the dataset identifier PXD050199 and 10.6019/PXD050199. The data generated in this study are provided in the Source Data file.

Research involving human participants, their data, or biological material

Policy information about studies with human participants or human data. See also policy information about sex, gender (identity/presentation), and sexual orientation and race, ethnicity and racism.

Reporting on sex and gender	Urinary catheters and urine samples were collected from both male and female catheterized patients. Urine samples were also collected from healthy female donors.
Reporting on race, ethnicity, or other socially relevant groupings	Urine samples form healthy female donors included people from different ethnicities such as Caucasian, Asian, Latino, and Black.<br>Ethnicity information from the catheterized patient was not available.
Population characteristics	Catheterized patients were both male and female from undisclosed ages in accordance with the IRB. Healthy and non-catheterized donors ranged from ages 24 to 45 and were female only to match female-only mice data for bacterial protease experiments. Donor health was indicated through lack of kidney disease, diabetes mellitus, or recent antibiotic treatment. Healthy donors were volunteers.
Recruitment	Patient catheters were collected with informed consent after the clinical decision to remove for standard of care was made by medical care.
Ethics oversight	This study was approved by the Washington University School of Medicine (WUSM) Internal Review Board (approval #201410058) and performed in accordance with WUSM's ethical standards and the 1964 Helsinki declaration and its later amendments.

Note that full information on the approval of the study protocol must also be provided in the manuscript.

Field-specific reporting

Please select the one below that is the best fit for your research. If you are not sure, read the appropriate sections before making your selection.

☒ Life sciences ☐ Behavioural & social sciences ☐ Ecological, evolutionary & environmental sciences

For a reference copy of the document with all sections, see nature.com/documents/nr-reporting-summary-flat.pdf

Life sciences study design

All studies must disclose on these points even when the disclosure is negative.

Sample size	Sample sizes were a total of three independent experiments with n = 3-6 mice depending on the genotype. Three experimental repetitions were used to verify changes in inoculum during mouse experiments. Minimum of 5 replicates were needed to perform statistical analysis and determine significance, if any.
Data exclusions	For mice experiments, animals that lost the catheter were not included in this work.
Replication	Mouse experiments were successfully repeated at least three times to validate reproducibility. Replications are noted in figure legends.
Randomization	C57BL/6 mice used for TXA experiments were randomly assigned. Randomization was not relevant for mice experiments involving mice with mutations in the fibrin formation or fibrinolytic pathway since the infectious agent were kept consistent between compared groups.
Blinding	The investigators involved in cytokine analyses and proteomic analyses were blinded and not involved with data analysis. All mouse model of infections were blinded in regards to the investigators involved in infections were not also involved in sample acquisition or data analysis.

Reporting for specific materials, systems and methods

We require information from authors about some types of materials, experimental systems and methods used in many studies. Here, indicate whether each material, system or method listed is relevant to your study. If you are not sure if a list item applies to your research, read the appropriate section before selecting a response.

Materials & experimental systems

n/a	Involved in the study
☐	☒ Antibodies
☒	☐ Eukaryotic cell lines
☒	☐ Palaeontology and archaeology
☐	☒ Animals and other organisms
☐	☒ Clinical data
☒	☐ Dual use research of concern
☒	☐ Plants

Methods

n/a	Involved in the study
☒	☐ ChIP-seq
☒	☐ Flow cytometry
☒	☐ MRI-based neuroimaging

Antibodies

Antibodies used

Antibodies used for western blot analysis were commercially available and include:
Anti-beta-Actin (Abcam ab8229)
Anti-fibrinogen (Abcam ab34269)
Donkey Anti-Goat IgG Polyclonal Antibody (LI-COR Biosciences 925-68074)
Donkey Anti-Rabbit IgG Polyclonal Antibody (LI-COR Biosciences 926-32213)
Plasminogen polyclonal antibody (Proteintech 17462-1-AP)
Thrombin polyclonal antibody (Fisher-Scientific PA5-99213)

Validation

Commercially used antibodies were validated by the manufactures as stated on the manufacturer websites with relevant citations and antibody profile, including:
Anti-beta-Actin (https://www.abcam.com/products/primary-antibodies/beta-actin-antibody-loading-control-ab8229.html)
Anti-fibrinogen (https://www.abcam.com/products/primary-antibodies/fibrinogen-antibody-ab34269.html)
Donkey Anti-Goat IgG Polyclonal Antibody (https://www.licor.com/bio/reagents/irdye-680rd-donkey-anti-goat-igg-secondaryantibody)
Donkey Anti-Rabbit IgG Polyclonal Antibody (https://www.licor.com/bio/support/contents/reagents/irdye-secondaryantibodies/800cw/donkey-anti-rabbit-igg.html)
Plasminogen polyclonal antibody (https://www.ptglab.com/products/PLG-Antibody-17462-1-AP.htm)
Thrombin polyclonal antibody (Fisher-Scientific PAS-99213)

Animals and other research organisms

Policy information about studies involving animals; ARRIVE guidelines recommended for reporting animal research, and Sex and Gender in Research

Laboratory animals

Mice used in this study were — ~6-week-old C57BL/6-background mice purchased from Jackson Laboratory and National Institute of Cancer Research or mutant mice bred in Freimann Life Science Center (Notre Dame, Indiana). Mice were housed in normal housing conditions with 12-hour light/dark cycles, 45-65% humidity, 20-24 C ambient temperature.

Wild animals

No wild animals were used in this study.

Reporting on sex

Mice used in this study were only healthy female mice due to the urogenital anatomy anatomy of the female mice permitting a urinary catheter. Male mice urogenital anatomy has many orifices that likely induce unintended damage pain to male mice. Thus, only female mice were used for this study.

Field-collected samples

No samples were field-collected.

Ethics oversight

The University of Notre Dame Institutional 443 Animal Care and Use Committee approved all mouse infections and procedures as part of protocol 444 number 22-01-6971. All animal care was consistent with the Guide for the Care and Use of 445 Laboratory Animals from the National Research Council. For urine collection for bacterial assays, all participants signed an informed consent form and protocols were approved by the local Internal Review Board at the University of Notre Dame under study #19-04-5273.

Note that full information on the approval of the study protocol must also be provided in the manuscript.

Clinical data

Policy information about clinical studies
All manuscripts should comply with the ICMJE guidelines for publication of clinical research and a completed CONSORT checklist must be included with all submissions.

Clinical trial registration

N/A

Study protocol	N/A
Data collection	N/A
Outcomes	N/A

Plants

Seed stocks	N/A
Novel plant genotypes	N/A
Authentication	N/A
